# Supplementary material for: Dynalign II: common secondary structure prediction for RNA homologs with domain insertions
Source: Nucleic Acids Res. 2014 Nov 21;42(22):13939–48. doi: 10.1093/nar/gku1172 (PMC4267632; doi:10.1093/nar/gku1172)
Supplement: SUPPLEMENTARY DATA [file supp_gku1172_nar-02021-z-2014-File012.zip › manual/javadoc/RNAstructure_java_utilities/MacChecker.html]

MacChecker


JavaScript is disabled on your browser.


- Overview
- Package
- Class
- Tree
- Index
- Help

- Prev Class
- Next Class

- Frames
- No Frames

- All Classes

- Summary:
- Nested |
- Field |
- Constr |
- Method

- Detail:
- Field |
- Constr |
- Method


RNAstructure\_java\_utilities

## Class MacChecker

- java.lang.Object
- - RNAstructure\_java\_utilities.MacChecker

- All Implemented Interfaces:
  :   Serializable

  ---

    

  ```
  public class MacChecker
  extends Object
  implements Serializable
  ```

  A class that handles checking whether an OS is a Mac, and setting general
  properties if that is the case.

  See Also:
  :   Serialized Form

- - ### Field Summary

    Fields

    | Modifier and Type | Field and Description |
    | --- | --- |
    | `private static long` | `serialVersionUID` |
  - ### Constructor Summary

    Constructors

    Constructor and Description

    `MacChecker()`
  - ### Method Summary

    Methods

    | Modifier and Type | Method and Description |
    | --- | --- |
    | `static void` | `checkOS()` Check the OS to see if it's a Mac, and if it is, set a few system properties to make things more "Mac-like." |
    | `static boolean` | `isMac()` Get whether the current OS is a Mac. |
    | `static boolean` | `notMac()` Get whether the current OS is not a Mac. |

    - ### Methods inherited from class java.lang.Object

      `clone, equals, finalize, getClass, hashCode, notify, notifyAll, toString, wait, wait, wait`

- - ### Field Detail


    - #### serialVersionUID

      ```
      private static final long serialVersionUID
      ```

      See Also:
      :   Constant Field Values
  - ### Constructor Detail


    - #### MacChecker

      ```
      public MacChecker()
      ```
  - ### Method Detail


    - #### checkOS

      ```
      public static void checkOS()
      ```

      Check the OS to see if it's a Mac, and if it is, set a few system
      properties to make things more "Mac-like."


    - #### isMac

      ```
      public static boolean isMac()
      ```

      Get whether the current OS is a Mac.

      Returns:
      :   True if the OS is a Mac, false if not.


    - #### notMac

      ```
      public static boolean notMac()
      ```

      Get whether the current OS is not a Mac.

      Returns:
      :   True if the OS is not a Mac, false if it is.


- Overview
- Package
- Class
- Tree
- Index
- Help

- Prev Class
- Next Class

- Frames
- No Frames

- All Classes

- Summary:
- Nested |
- Field |
- Constr |
- Method

- Detail:
- Field |
- Constr |
- Method
